# Idiopathic Retroperitoneal Fibrosis Presenting as Low Back Pain

**DOI:** 10.7759/cureus.4075

**Published:** 2019-02-14

**Authors:** Shorabh Sharma, Julian A Paniagua-Morales, Giovanni Gerardo Cordoba, Eric O Then, Sapna Sharma

**Affiliations:** 1 Internal Medicine, St. Barnabas Hospital Health System, Bronx, USA; 2 Internal Medicine, Mahatma Gandhi Mission Institute of Health Sciences, Navi Mumbai, USA

**Keywords:** idiopathic retroperitoneal fibrosis, hydronephrosis, ormonds disease

## Abstract

Retroperitoneal fibrosis is a rare condition characterized by the presence of fibrous inflammatory tissue in the retroperitoneal structures, such as the infrarenal great vessels and ureters. We are reporting an atypical case of an alcoholic who presented with chronic back pain and abnormal liver function tests. Abdominal imaging revealed an incidental, ill-defined, abnormal soft tissue mass in the left pelvis and mild to moderate left hydroureteronephrosis. Interventional radiology (IR)-guided core biopsy reported cores of dense fibrous tissue with extensive lymphoplasmacytic infiltrates, consistent with idiopathic retroperitoneal fibrosis (IRF). The patient had a left ureteral stent placed and, subsequently, had robotic surgery for ureteral reimplantation.

## Introduction

Retroperitoneal fibrosis is a rare condition characterized by the presence of fibrous inflammatory tissue in retroperitoneal structures. This condition is generally idiopathic in origin but may have secondary causes. Most cases present with flank pain as the main symptom and new-onset hypertension as the most common sign. Prompt recognition and early treatment are important since chronic renal failure and disease relapse may occur [1-3]. We present a case of retroperitoneal fibrosis where the patient was found to have renal complications with chronic low back pain as the only symptom.

## Case presentation

**Figure 1:**
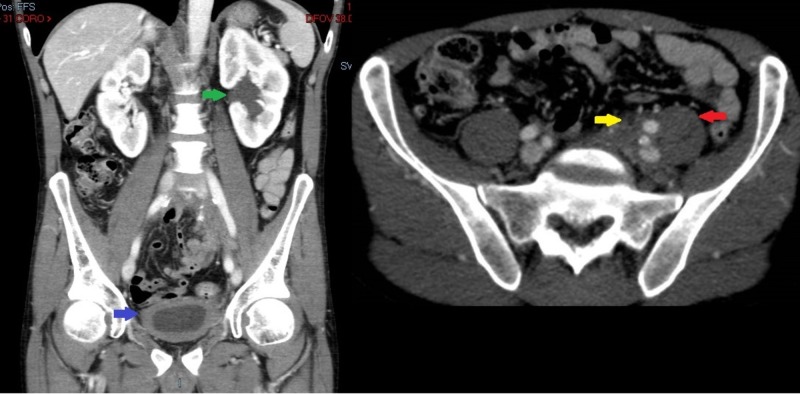
Axial and coronal computerized tomography images demonstrating left-sided pelvic mass causing hydroureteronephrosis (green arrow) with diffuse bladder wall thickening (blue arrow). Asymmetric pelvic mass extending to posterior presacral region and psoas (red arrow), with ureter encased by pelvic mass (yellow arrow).

A 50-year-old African-American male with a medical history of hypertension, alcohol and marijuana use disorder, with macrocytic anemia and transaminitis, presented to our clinic to establish care. He reported a history of chronic back pain for which he took over-the-counter pain medications. He was also taking amlodipine for his hypertension. He was referred to gastroenterology for a screening colonoscopy; autoimmune and viral work-up for transaminitis were negative. An abdominal ultrasound was obtained for the transaminitis and revealed a slight fullness of the left renal collecting duct, and computed tomography of the abdomen and pelvis was suggested. The latter (Figure 1) revealed an asymmetric, ill-defined soft tissue mass in the left pelvic inlet and sidewall involving the distal left ureter and seminal vesicle and prostate, producing mild left hydronephrosis, hydroureter, and a left, retroperitoneal, 1.7 cm, enlarged lymph node. CT with contrast revealed an ill-defined abnormal soft tissue in the left pelvis extending to the left pelvic sidewall and posterior presacral region concerning for neoplasm and mild to moderate left hydroureteronephrosis secondary to the encasement of the distal left ureter by left pelvic soft tissue.

Oncology was consulted, and he was referred for a biopsy. An interventional radiology-guided core biopsy was performed, which reported cores of dense fibrous tissue with extensive lymphoplasmacytic infiltrates. He was referred to urology, had a left ureteral stent placed, and, subsequently, had robotic surgery for ureteral stent reimplantation.

## Discussion

Retroperitoneal fibrosis, formerly known as Ormond’s disease, is a rare condition characterized by the presence of fibrous inflammatory tissue in retroperitoneal structures. Causes include certain drugs, malignant diseases, infections, or surgery although, over 70% of cases are of idiopathic origin and are either immunoglobulin G4 (IgG4) or non-IgG4 related [1-3].

The incidence of the idiopathic form of the disease is estimated to be 1.3 per 100,000 inhabitants per year [4]. Studies have suggested a male to female predominance, with most patients being in the early 50s [2,5].

Studies have reported pain usually in one or both flanks with radiation to the inguinal area as the main symptom (94%) and new-onset hypertension as the most common sign (33%); patients may also present with fatigue, anorexia, weight loss, fever, hydroceles, scrotal pain, lower extremity edema, and pulmonary embolism. Since ureters may be affected, various degrees of ureteral obstruction, hydronephrosis, and renal failure are also considered early and common clinical manifestations. Abdominal pain and obstructive uropathy should, therefore, raise suspicion for retroperitoneal fibrosis and imaging should be obtained [2,6].

If there is no clear etiology suspected and idiopathic retroperitoneal fibrosis is presumed; it is still reasonable to obtain antinuclear antibodies (ANAs), immunoglobulin G4 (IgG4), anti-smooth muscle antibodies, antinuclear cytoplasmic antibodies (ANCAs), thyroid function tests, and antibodies against thyroid microsomal and thyroglobulin. ANA has been reported positive in up to 50% of cases while anti-thyroid microsome and thyroglobulin are positive in approximately one-fourth of the cases, suggesting autoimmune thyroiditis. Moreover, a positive ANCA has been described in cases associated with granulomatosis with polyangiitis and microscopic polyangiitis [7-8]. Imaging and/or biopsy often provide the diagnosis. CT scan, magnetic resonance imaging (MRI), and renal ultrasonography are usually performed; the latter is often the first imaging study performed since most cases present with obstructive urinary symptoms. A CT scan has the additional advantage of enabling CT-guided biopsy to obtain tissue for pathologic analysis, whereas MRI may provide a better definition of retroperitoneal fibrosis against the surrounding tissues and may help differentiate malignant causes in T2-weighted scans. A definitive diagnosis, however, may require a biopsy to confirm the diagnosis and/or exclude secondary causes like malignancy [9].

Treatment of the underlying etiology or discontinuation of the offending drug is done when retroperitoneal fibrosis is due to a secondary cause, as mentioned above. Medical treatment for idiopathic retroperitoneal fibrosis is used for stopping the progression of the fibrotic process and preventing recurrence. Moreover, since most patients present with urinary obstruction symptoms caused by fibrosis, relief of obstruction by the percutaneous, open surgical, or endoureteral approach is advised, especially in patients with renal function compromise [1,10].

Therapy with glucocorticoids like methylprednisolone or prednisone is considered the mainstay of treatment. The recommended dose of prednisone is 1 mg/kg per day (maximum dose: 80 mg/day) for four weeks. If improvement is observed, it can be tapered over two to three months to 10 mg/day and maintained for an additional six months. If there is no response to initial glucocorticoid therapy, a combination with immunosuppressives like azathioprine, mycophenolate mofetil, methotrexate, cyclophosphamide, and cyclosporine can be considered. Tamoxifen has also been used in patients with contraindications to steroid use or in those who do not tolerate steroid treatment. However, prednisone has shown to be more effective in preventing relapses when compared to tamoxifen. In cases of immunoglobulin G4 (IgG4)-related retroperitoneal fibrosis, there have been case reports in which the spontaneous remission of clinical symptoms and radiological abnormalities occurred [11-15].

In our patient’s first encounter to establish primary care, he had been off antihypertensives for well-controlled blood pressure for the previous four years and endorsed a history of chronic low back pain. The chemistry panel revealed a serum creatinine of 1.3. Immunologic workup revealed positive anti-smooth muscle antibody in 1:40 titer and negative antinuclear antibodies. In addition to the CT abdomen and pelvis findings described above, there were also multiple pelvic vascular calcifications but no aortic or renovascular involvement. Therefore, it is unclear if his uncontrolled hypertension was related to worsening retroperitoneal fibrosis. Moreover, he endorsed no urinary symptoms other than ill-defined flank pain in spite of a significant hydroureter finding in CT imaging. Prior to surgical intervention, there was an improvement of serum creatinine to 1.0. Free Kappa and Lambda chains were present in urine in a 14.99 Kappa/Lambda ratio, along with moderate macrocytic anemia. B2 microglobulin, urine, and serum protein electrophoresis showed no abnormality. The patient was later lost to follow-up for the further evaluation of a possible plasma cell disorder.

## Conclusions

In retrospect, IRF is an extremely rare pathology with varying degrees of severity. It is important to keep this differential diagnosis in mind when encountering patients who present with low back or flank pain, newly diagnosed or uncontrolled hypertension, and urinary complaints. This is especially true in patients with alcohol use disorder, where clinicians oftentimes commit anchoring biases toward the diagnosis of alcoholic pancreatitis. After obtaining surgical biopsy confirmation, IRF should be treated promptly with glucocorticoids or immunosuppressive agents to reduce the risk of complications. These include worsening hypertension, hydronephrosis, and renal failure among others.
